# Validation of the NoSAS Score for the Screening of Sleep-Disordered Breathing in a Sleep Clinic

**DOI:** 10.1155/2020/4936423

**Published:** 2020-01-08

**Authors:** Yi Rong, Shihan Wang, Hui Wang, Feng Wang, Jingjing Tang, Xiuhong Kang, Guangxi Li, Zhiguo Liu

**Affiliations:** ^1^Guang'anmen Hospital, Chinese Academy of Chinese Medical Sciences, Beijing 100053, China; ^2^School of Management, Beijing University of Chinese Medicine, Beijing 100029, China; ^3^Department of Respiratory and Critical Care Medicine, Beijing Chaoyang Hospital, Beijing Institute of Respiratory Medicine and Capital Medical University, Beijing 100020, China

## Abstract

**Background:**

There is a growing number of patients with sleep-disordered breathing (SDB) referred to sleep clinics. Therefore, a simple but useful screening tool is urgent. The NoSAS score, containing only five items, has been developed and validated in population-based studies.

**Aim:**

To evaluate the performance of the NoSAS score for the screening of SDB patients from a sleep clinic in China, and to compare the predictive value of the NoSAS score with the STOP-Bang questionnaire.

**Methods:**

We enrolled consecutive patients from a sleep clinic who had undergone apnea-hypopnea index (AHI) testing by type III portable monitor device at the hospital and completed the STOP-Bang questionnaire. The NoSAS score was assessed by reviewing medical records. Sensitivity, specificity, positive predictive value, negative predictive value, and area under the receiver operating characteristic curve (AUC) of both screening tools were calculated at different AHI cutoffs to compare the performance of SDB screening.

**Results:**

Of the 596 eligible patients (397 males and 199 female), 514 were diagnosed with SDB. When predicting overall (AHI ≥ 5), moderate-to-severe (AHI ≥ 15), and severe (AHI ≥ 30) SDB, the sensitivity and specificity of the NoSAS score were 71.2, 80.4, and 83.1% and 62.4, 49.3, and 40.7%, respectively. At all AHI cutoffs, the AUC ranged from 0.688 to 0.715 for the NoSAS score and from 0.663 to 0.693 for the STOP-Bang questionnaire. The NoSAS score had the largest AUC (0.715, 95% CI: 0.655–0.775) of diagnosing SDB at AHI cutoff of ≥5 events/h. NoSAS performed better in discriminating moderate-to-severe SDB than STOP-Bang with a marginally significantly higher AUC (0.697 vs. 0.663, *P*=0.046).

**Conclusion:**

The NoSAS score had good performance on the discrimination of SDB patients in sleep clinic and can be utilized as an effective screening tool in clinical practice.

## 1. Introduction

Sleep-disordered breathing (SDB) is featured by recurrent obstruction of the upper airway during sleep, affecting 2–26% of the general population [1]. Patients with untreated SDB are more predisposed to other morbidities, including cardiovascular diseases [2], postoperative complications [3], and traffic accidents [4]. It is necessary to screen SDB accurately to identify patients at a high risk, for the sake of prompt treatment and prevention of other morbidities. In-laboratory polysomnography (PSG) is the gold standard of SDB diagnosis, but it is time-consuming and expensive that has limited the accessibility.

In recent years, several simple, costless, and acceptant screening tools based on clinical symptoms and risk factors have been developed to assist in the classification of low-risk or high-risk SDB patients [5, 6]. The STOP-Bang questionnaire, an 8-item tool, has been validated to be useful in screening SDB in preoperative patients [6]. It includes 8 yes/no questions and ranges from 0 to 8 scores. At a cutoff score of 3, the STOP-Bang questionnaire can detect moderate and severe SDB with a high sensitivity of 92.9% and 100%, respectively. Compared with other scoring models such as Berlin questionnaire and Epworth Sleeping Scale (ESS), STOP-Bang questionnaire usually has a better performance in various populations [7, 8]; therefore, it is gradually widely used in sleep disorder clinics.

Recently, a new screening tool named NoSAS score was developed and validated in a population-based investigation [9]. The NoSAS score ranges from 0 to 17 points and allocates different points to 5 items. At a threshold of 8 points, it performed significantly better than STOP-Bang and Berlin questionnaires with a larger area under the ROC curve (AUC), indicating that NoSAS score was an efficient tool to discriminate individuals at high risk of SDB. Some studies performed comparisons between the NoSAS score and other questionnaires in different populations and found similar or better performances of NoSAS score to STOP-Bang, Berlin, or ESS questionnaires [10–12].

However, most of these studies were performed in the general population [9, 10] or hospital-based cohorts [11]. These populations may differ from subjects recruited from the sleep clinics who sought for diagnosis or treatment due to sleeping disorders. Therefore, the performance of the NoSAS score in predicting SDB in sleep clinics needs further investigation. In the present study, we validated and compared the performance of the NoSAS score and STOP-Bang questionnaire in screening SDB patients in a sleep clinic.

## 2. Materials and Methods

### 2.1. Participant Recruitment

All participants were recruited from the sleep clinic of Guang'anmen Hospital, China Academy of Chinese Medical Science, Beijing, China, from January 2015 to September 2017. Anyone who did not complete questionnaire, refused monitoring, had total sleeping time <4 hours, was younger than 18 years old, or was diagnosed as SDB previously was excluded. The characteristics of each participant, including gender, age, weight, height, neck and waist circumferences, self-reported hypertension, smoking status, were measured or documented. This study was approved by the Ethics Committee of Guang'anmen Hospital, and all the participants gave written informed consents.

### 2.2. The NoSAS Score

The NoSAS score allocates 4 points for having a neck circumference >40 cm, 3 points for having a body mass index (BMI) between 25 and <30 kg/m^2^ or 5 points for having a BMI ≥ 30 kg/m^2^, 2 points for snoring, 4 points for being older than 55 years, and 2 points for being male. The threshold was set as 8 points. The NoSAS score was assessed by retrospectively reviewing the medical records of each participant.

### 2.3. STOP-Bang Questionnaire

We applied the Chinese version of the STOP-Bang questionnaire originally developed by Chung et al. [6]. Briefly, it contains 8 items related to loud snoring, tiredness during daytime, observed apnea during sleep, high blood pressure, age, neck circumference, body mass index (BMI), and gender. One point will be given for answering “yes” and zero score for “no.” If one answered yes in three or more items, he/she was considered to have a high risk of SDB. Notably, we applied 30 kg/m^2^ as the BMI threshold instead of 35 kg/m^2^, which was previously suggested by Ong et al. [13]. STOP-Bang questionnaire was completed by the participants themselves.

### 2.4. Portable Monitoring

After finishing a STOP-Bang questionnaire, the participants were invited to undergo type III portable monitor device (ApneaLink device; ResMed, Sydney, Australia). Yet, the monitoring was performed by the clinicians in the hospital instead of by the patients themselves at home to ensure the accuracy of operations. We recorded thoracoabdominal movement, nasal airflow, pulse oximetry, and snoring. Sleep-related breathing events were evaluated by researchers who were blinded to the results of the STOP-Bang questionnaire according to the guideline released by the American Academy of Sleep Medicine [14]. Apnea was defined as a ≥ 90% reduction of airflow from baseline for at least 10 seconds, while hypopnea was defined as ≥30% decrease of airflow lasting at least 10 seconds, associated with either an arousal or a ≥ 3% oxygen saturation decrease [14]. AHI (apnea-hypopnea index) was calculated as the mean number of apnea and hypopnea events per hour of sleep. SDB was diagnosed as AHI ≥ 5 events/h, and the severity was defined as follows: AHI ≥ 5 and <15 events/h for mild SDB, AHI ≥ 15 and <30 events/h for moderate SDB, and AHI ≥ 30 events/h for severe SDB.

### 2.5. Statistics

All the statistical analyses were performed by SPSS v.19 (SPSS Inc., Chicago, Illinois, USA). Student's *t*-test, chi-square test, and Mann–Whitney *U* test were used for the comparison between groups for means, frequencies, and medians, respectively. Sensitivity, specificity, positive predictive value (PPV), and negative predictive value (NPV) were calculated to assess the predictive values of the NoSAS score and STOP-Bang questionnaire in diagnosing SDB at different AHI cutoffs. Receiver operating characteristic curve (ROC) analysis was performed and the area under the ROC curve (AUC) was calculated. DeLong test [15] was employed to compare the AUCs of both screening tools. *P* value < 0.05 was considered statistically significant.

## 3. Results

### 3.1. Characteristics of All Participants

A total of 667 individuals were consecutively recruited, of whom 12 refused monitoring, 13 had insufficient recording time of <4 hours, 8 had incomplete STOP-Bang questionnaire, 18 were younger than 18 years, and 20 had previously diagnosed SDB. Finally, 596 (89.4%) individuals met the inclusion criteria and enrolled for the present study. Among them, 514 patients were diagnosed as SDB (AHI ≥ 5 events/h) and 82 were non-SDB (AHI < 5 events/h) by portable polysomnography monitoring. SDB was found in 86.2% of the patients and the prevalence of mild, moderate, and severe SDB was 33.2%, 23.2%, and 29.9%, respectively.

There were 397 males and 199 females. Overall, mean values (±SD) for age, BMI, and neck circumference were 54.4 ± 13.9 years, 27.4 ± 4.0 kg/m^2^, and 39.1 ± 3.9 cm, respectively. In the SDB group, 27.2%, 46.1%, and 26.6% of participants had BMI <25, 25 to <30, or ≥30 kg/m^2^, respectively, which differed significantly from that of the non-SDB group (*P*=0.011). There was higher frequency of individuals with >40 cm circumference in the SDB group than in the non-SDB group (41.2% vs. 25.6%, *P*=0.007). Additionally, we observed significant differences in age, waist circumference, nadir SpO_2_, STOP-Bang score, the NoSAS score, and AHI, but not in gender, hypertension, and current smoking status, between the SDB and non-SDB groups. The characteristics of all patients are summarized in Table 1.

### 3.2. Predictive Values of Both Screening Tools

The diagnostic properties of the NoSAS score and STOP-Bang questionnaire, including sensitivity, specificity, PPV, and NPV, were calculated at different AHI cutoffs (≥5, 10, 15, 20, 25, and 30) as shown in Table 2. At the AHI cutoff of ≥5 events/h and using a threshold of ≥8 scores, the sensitivity, specificity, PPV, and NPV of NoSAS score to predict SDB were 71.2%, 62.4%, 92.4%, and 26.2%, respectively. As the AHI cutoffs increased from 5 to 30 events/h, the sensitivity of the NoSAS score increased from 71.2% to 83.1% and NPV from 26.0% to 85.0%. Meanwhile, there were reducing trends of specificity from 62.4% to 40.7% and PPV from 92.4% to 37.4%. Comparing both screening tools in predicting SDB at various AHI cutoffs, we found that the NoSAS scores had higher specificity and PPV but lower sensitivity and NPV than the STOP-Bang questionnaire.

**Figure 1:**
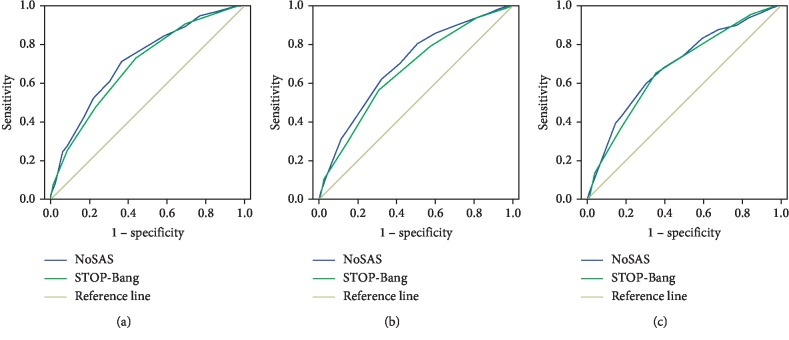
ROC curves of the NoSAS score and STOP-Bang questionnaire in predicting SDB. (a) At the AHI cutoff of ≥5 events/h; (b) at the AHI cutoff of ≥15 events/h; (c) at the AHI cutoff of ≥30 events/h. ROC: receiver operating characteristics; SDB: sleep-disordered breathing; AHI: apnea-hypopnea index.

Figure 1 shows the discriminatory abilities of both tools in screening all (Figure 1(a)), moderate-to-severe (Figure 1(b)), and severe SDB (Figure 1(c)), respectively. The AUCs ranged from 0.685 to 0.715 for the NoSAS score and from 0.663 to 0.693 for the STOP-Bang questionnaire. When discriminating moderate-to-severe SDB, the NoSAS score performed better than the STOP-Bang questionnaire that reached marginal significance (AUC: 0.697 vs. 0.663, *P*=0.043), whereas the performance of both tools did not differ significantly at the other levels of SDB severity (Table 2).

## 4. Discussion

In the present study, we aimed at assessing the clinical utility of the NoSAS score in a population from the sleep clinic where there was a high prevalence of SDB (86.2%). We found the NoSAS score had discriminatory power for SDB screening with AUCs clustering around 0.685–0.715 at various AHI cutoffs. Our results suggested a potential application of the NoSAS score in screening SDB in clinical settings.

The NoSAS score was firstly developed as a simple but efficient tool for SDB screening in a population-based study [9]. Compared with the other tools including Berlin, ESS, and STOP-Bang questionnaires, it contains limited number of items. Among the five variables of the NoSAS score, the age and gender are very objective, and the BMI and neck circumference can be easily and accurately measured. Therefore, it is very convenient for clinicians and participants to implement this tool in SDB screening and the value has been validated in various populations in comparison with other screening tools [10–12, 16, 17].

In two population-based studies, one in the Caucasian population [9] and one in the Asian population [10], the NoSAS score was found to perform better to discriminate SDB and non-SDB than did the STOP-Bang questionnaire, especially in those with higher SDB severity. The AUCs of the NoSAS score reached as high as 0.81 in the EPISONO cohort and 0.748 in the Asian population while the AUCs of STOP-Bang were almost around 0.7. When applied in hospital-based populations [11, 12], the NoSAS score had equivalent or higher performance than the other tools including STOP-Bang questionnaire, ESS, and Berlin scores. Additionally, the NoSAS score was shown to be effective in predicting SDB in patients with depressive majors [16] or with insomnia [17]. In the present study, we compared the performance of the NoSAS score to the STOP-Bang questionnaire in a sleep clinic-based population. Despite the differences in sensitivity and specificity between both tools, the NoSAS score seems to have better sensitivity and specificity compromise than STOP-Bang questionnaire at some AHI cutoffs. We found that the NoSAS score performed significantly better when discriminating moderate-to-severe SDB, whereas both tools had similar AUCs at the other AHI cutoffs. These investigations and validations have proved the NoSAS score as a simple but efficient screening tool for SDB in these populations.

Our study, performed in a population from a sleep clinic, yielded an obvious lower AUC of the NoSAS score than a previous study [18] which included Caucasians and was also carried out in a sleep clinic (0.715 vs. 0.770 for all SDB; 0.697 vs. 0.746 for moderate-to-severe SDB). Similar results were also observed that AUCs of the NoSAS score in the Asian populations [10–12] were lower than in the Caucasian populations [9, 16, 17]. These may be partially explained by the difference in the pathogenesis of SDB between Asians and Caucasians. It has been proposed that craniofacial factors or arousal threshold may contribute more than obesity to the development of SDB in Chinese [19, 20]. Previous data showed that the Asians may develop SDB even at lower BMI [21], which was also supported by our data that 27.2%, 46.1%, and 26.6% of SDB were at BMIs of <25, 25 to <30, and ≥30 kg/m^2^, respectively, compared to 5.7%, 41.3%, and 53.0% in Caucasians [18]. Therefore, BMI may not present an indicator in predicting SDB in Asians as good enough as in Caucasians. However, the NoSAS score still had good discriminatory ability and performed better than the STOP-Bang questionnaire in Chinese populations.

Our study had some limitations. Firstly, this was a retrospective study. The STOP-Bang questionnaire was completed before monitoring, but the NoSAS score was obtained by reviewing medical records. Although the NoSAS score is simple enough and the “snoring” item, which is different from the “loud snoring” item in the STOP-Bang questionnaire, can be collected from medical records, it may still introduce some bias. However, we found that all individuals reporting loud snoring and someone being negative for this item in the STOP-Bang questionnaire were positive for “snoring” item in the NoSAS score. In our final analysis, 385 (64.6%) individuals reported loud snoring in the STOP-Bang questionnaire, while 513 (86.1%) were positive for “snoring” item of the NoSAS score. Considering the high percentage of snoring in our study, the bias that some snorers may not have been recorded and retrospectively collected should be limited. Secondly, the participants were enrolled from a single sleep center who the referred due to sleep-related problems. There was a high prevalence of SDB in our study. It would influence the accuracy of this screening tool and limit the interpretation and application of our results to the general populations. Thirdly, we diagnosed SDB based on type III portable monitor rather than in-laboratory polysomnography, the gold standard method for SDB diagnosis. A previous population-based study showed a good AHI concordance between portable monitors and polysomnography, but portable monitors tended to slightly underestimate AHI [22]. However, in the present study, portable monitoring was conducted in hospital by trained clinicians, instead of at home by the patients themselves, to ensure the accuracy of the testing and reducing the discordance.

## 5. Conclusion

In conclusion, we found that the NoSAS score had good predictive value for the screening of SDB in patients from a sleep clinic and can be used as an easy and effective screening tool in clinical practice.

## Figures and Tables

**Table 1:** Baseline characteristics of all participants.

Variables	Total	Non-SDB	SDB	*P* value^*∗*^
Number (*n*)	596	82	514	
Male *n* (%)	397 (66.6%)	51 (62.2%)	346 (67.3%)	0.361
Age (years)	54.4 (±13.9)	48.6 (±12.5)	55.3 (±13.9)	<0.001
BMI (kg/m^2^)	27.4 (±4.0)	26.1 (±3.9)	27.6 (±4.0)	<0.001
<25 kg/m^2^*n* (%)	174 (29.2%)	34 (41.5%)	140 (27.2%)	
25 to <30 kg/m^2^*n* (%)	273 (45.8%)	36 (43.9%)	237 (46.1%)	0.011^&^
≥30 kg/m^2^*n* (%)	149 (25.0%)	12 (14.6%)	137 (26.6%)	
Neck circumference (cm)	39.1 (±3.9)	37.6 (±3.8)	39.4 (±3.9)	<0.001
≤40 cm *n* (%)	363 (60.9%)	61 (74.4%)	302 (58.8%)	0.007^#^
>40 cm *n* (%)	233 (39.1%)	21 (25.6%)	212 (41.2%)	
Waist circumference (cm)	98.8 (±18.6)	94.5 (±11.9)	99.6 (±19.3)	<0.001
Hypertension *n* (%)	356 (59.7%)	47 (57.3%)	309 (60.1%)	0.632
Current smokers *n* (%)	176 (30.0%)	22 (26.8%)	154 (30.0%)	0.564
STOP-Bang score	4.3 (±1.5)	3.4 (±1.5)	4.4 (±1.4)	<0.001
NoSAS score	9.0 (±3.7)	6.5 (±3.6)	9.4 (±3.6)	<0.001
Nadir SpO_2_ (%)	83.1 (±10.2)	89.9 (±2.7)	80.7 (±10.8)	<0.001
AHI (events/hour)	16.0 (7.3–35.2)	2.6 (1.5–3.8)	19.3 (10.5–38.5)	<0.001

Age, BMI, neck circumference, waist circumference, nadir SpO_2_, STOP-Bang score, and NoSAS score are presented as mean ± standard deviation. AHI is presented as median and interquartile range. OSA: sleep-disordered breathing; AHI: apnea-hypopnea index; BMI: body mass index; SDB is defined as AHI ≥ 5 events/h by polysomnography.^*∗*^Non-SDB versus SDB. ^&^*P* value of chi-square test of BMI subgroups.^#^*P* value of chi-square test of neck circumference subgroups.

**Table 2:** Predictive values of NoSAS score and STOP-Bang questionnaire at different AHI cutoffs.

Screening tools	Sensitivity (95% CI)	Specificity (95% CI)	PPV (95% CI)	NPV (95% CI)	AUC (95% CI)	*P* ^*∗*^
AHI ≥ 5						0.330
NoSAS	71.2% (87.7–93.0)	62.4% (52.0–73.6)	92.4% (89.2–94.7)	26.0% (20.2–32.8)	0.715 (0.655–0.775)	
STOP-Bang	90.7% (87.7–93.0)	30.5% (21.0–41.8)	89.1% (86.0–91.6)	34.4% (23.8–46.4)	0.693 (0.631–0.755)	
AHI ≥ 10						0.072
NoSAS	76.7% (72.2–80.7)	53.7% (46.6–60.7)	86.5% (72.0–80.5)	54.0% (46.8–61.0)	0.705 (0.661–0.749)	
STOP-Bang	92.9% (89.9–95.2)	22.4% (17.0–28.9)	70.2% (66.0–74.0)	61.6% (49.5–72.5)	0.672 (0.627–0.718)	
AHI ≥ 15						0.046
NoSAS	80.4% (75.5–84.5)	49.3% (43.4–55.3)	64.1% (59.2–68.8)	69.0% (62.0–75.2)	0.697 (0.655–0.739)	
STOP-Bang	93.7% (90.2–96.0)	18.9% (14.6–24.1)	56.6% (52.2–60.9)	72.6% (60.7–82.1)	0.663 (0.619–0.706)	
AHI ≥ 20						0.165
NoSAS	81.4% (75.8–85.9)	44.1% (38.9–49.5)	50.8% (45.7–55.8)	77.0% (70.4–82.5)	0.697 (0.654–0.739)	
STOP-Bang	95.1% (91.4–97.3)	17.5% (13.7–22.0)	44.9% (40.7–49.3)	83.6% (72.6–90.8)	0.671 (0.627–0.714)	
AHI ≥ 25						0.264
NoSAS	82.6% (76.6–87.4)	42.2% (37.2–47.2)	43.2% (38.3–48.2)	82.0% (74.8–86.9)	0.685 (0.640–0.729)	
STOP-Bang	95.2% (91.0–97.5)	16.2% (12.8–20.3)	37.7% (33.5–42.0)	86.3% (75.8–92.9)	0.664 (0.618–0.709)	
AHI ≥ 30						0.512
NoSAS	83.1% (76.6–88.2)	40.7% (36.0–45.6)	37.4% (32.6–42.4)	85.0% (79.1–89.5)	0.688 (0.641–0.735)	
STOP-Bang	95.5% (91.0–97.9)	15.5% (12.3–19.5)	32.5% (28.5–36.7)	89.0% (79.0–94.8)	0.675 (0.628–0.722)	

^*∗*^ *P* value of DeLong test comparing AUCs of both screening tools. AHI: apnea-hypopnea index; PPV: positive predictive value; NPV: negative predictive value; AUC: area under the receiver operating characteristics curve.

## Data Availability

The data used to support the findings of this study are available from the corresponding author upon request.
